# Elder Mistreatment Victims during the COVID-19 Pandemic: Administrative Data from San Francisco Adult Protective Services

**DOI:** 10.1007/s10896-021-00305-1

**Published:** 2021-08-14

**Authors:** Pi-Ju Liu, Aining Wang, Laura M. Schwab-Reese, Sara K. Stratton

**Affiliations:** 1 School of Nursing and Center on Aging and the Life Course, Purdue University, 502 North University Street, West Lafayette, IN 47907 USA; 2 Department of Statistics, Purdue University, West Lafayette, USA; 3 Department of Public Health, Purdue University, West Lafayette, USA; 4 Department of Aging and Adult Services, San Francisco Adult Protective Services, San Francisco, USA

**Keywords:** Elder mistreatment, Abuse, Neglect, Exploitation, Self-neglect, Unmet need, Loneliness, Caregiving

## Abstract

This study examined elder mistreatment victims’ experiences at the beginning of the COVID-19 pandemic, focusing on their COVID-19 awareness and unmet needs. San Francisco Adult Protective Services (APS) caseworkers conducted phone interviews with clients or collaterals (client’s family, trusted other, or service provider) to inquire about clients’ awareness of COVID-19 and unmet needs. Nine-hundred-and-thirty-four (71%) of 1,313 APS’ past clients or their collaterals were interviewed, with 741 (79%) responding positively to COVID-19-awareness questions, and 697 (75%) having no unmet needs. Binary logistic regression with Firth adjusted maximum likelihood estimation method revealed that older persons (*p* < .05), self-neglectors (*p* < .05), and victims of neglect (*p* < .05) were less aware of COVID-19. Unmet needs varied by mistreatment type. Victims of isolation were more likely to have medical needs (*p* < .05), while victims of emotional abuse were more likely to report loneliness (*p* < .001). Case notes reflected clients who were well-prepared for the pandemic, versus those who required additional assistance to follow preventative measures of the COVID-19 pandemic to stay home. Although the majority of San Francisco APS’ past clients experienced no unmet needs at the beginning of the COVID-19 pandemic, the prolonged length and intensity of the pandemic could have exacerbated this vulnerable group’s situation. Collaboration between service providers is key in assisting victims experiencing unmet needs to live safely in a public health crisis, especially underserved victims of specific ethnic backgrounds.

## Introduction

The rapid outbreak of coronavirus disease 2019 (COVID-19) caused by the severe acute respiratory syndrome coronavirus 2 (SARS-CoV-2) has led to a global pandemic. Recommendations to intervene and prevent the spread of COVID-19, such as social distancing and self-quarantine, have drastically altered people’s lives (Luchetti et al., 2020). Older adults may be particularly impacted by the pandemic. Recent studies describe higher COVID-19 fatality rates among older adults with comorbidities (e.g., Le Couteur et al., 2020) and growing reports of elder mistreatment (Han & Mosqueda, 2020). In fact, elder abuse prevalence increased 83.6% at the beginning of the pandemic (Chang & Levy, 2021). Therefore, it may be necessary to attend to these age-related disparities during the remainder of the COVID-19 pandemic and future health crises.

The Abuse Intervention/Prevention Model (AIM), which integrates multiple factors associated with elder abuse and neglect, may inform approaches that mitigate risks of elder mistreatment during COVID-19, such as calling on local Adult Protective Services (APS) to adapt their services to protect older adults (Han & Mosqueda, 2020). This study examined San Francisco APS’ efforts in reaching out to their clients to provide COVID-19 education and identify unmet needs for referrals.

## Synergistic Risk of COVID-19 and Elder Mistreatment during the Pandemic

Older adults with otherwise heightened risk of mistreatment may also be more vulnerable to COVID-19. Some physical and cognitive symptoms that are more prevalent among elder mistreatment victims, such as hypertension and dementia (Heath et al., 2005), are also associated with higher COVID-19-related fatality rates, compared with individuals who do not have these comorbidities (Kuo et al., 2020; Schrack et al., 2020). Moreover, many elder mistreatment victims require home-based long-term care, which is another risk factor of COVID-19, specifically among those with comorbidities (Weng et al., 2020).

Beyond these shared risk factors, the COVID-19 pandemic response may further exacerbate poor health outcomes among individuals at risk for elder mistreatment. Although these responses, which varied by location, were intended to prevent the spread of COVID-19, they could lead to other negative consequences inadvertently. For example, it was common to require people to leave their homes for only essential activities, such as buying food or attending medical appointments. This requirement may negatively impact access to care and supplies, particularly among older adults who require specialized services and healthcare from professionals or family caregivers or have financial limitations (Makaroun et al., 2020). Obtaining appropriate nutrition may also be more challenging. Lack of proper nutrition may harm older adults’ physical and cognitive functioning and lead to unwanted weight gain or loss. Beyond adequate access to resources, the COVID-19 pandemic may challenge psychosocial functioning. Recently published studies found that older adults reported higher levels of loneliness, sadness, or depression when the COVID-19 outbreak began, and individuals who lived alone, had at least one chronic condition, or had reduced non-physical contacts, reported more negative experiences (Arpino et al., 2020; Luchetti et al., 2020). These challenges imposed by the COVID-19 pandemic, including anxiety of contracting the disease, changes in care routine, and loneliness due to lockdown, are associated with increased elder mistreatment risk (Schrack et al., 2020).

## Unmet Needs of Elder Mistreatment Victims

Elder mistreatment victims often need services, including healthcare, victim, legal, and financial services, from different providers to address abuse and other chronic or serious problems (Dauenhauer et al., 2019; Heath et al., 2005; Liu et al., 2021; Penhale, 2010). Based on a recent report from the National Adult Maltreatment Reporting System, the most common services directly provided to clients by APS were victim services, care/case management services, and in-home assistance services. In addition, the most common referrals for outside services included care/case management services, medical and dental services, burial/cremation, Alzheimer’s/dementia education, public health, animal control, and consultation (Aurelien et al., 2018).

Service needs of older adults at-risk of mistreatment have not been thoroughly investigated, but a recent study indicated older adults who experienced mistreatment have high levels of unmet service needs, including food, transportation, household services, housing, medical, and mental health (Olomi et al., 2019). Although the sample was restricted to older adults referred to a multidisciplinary team through police incident reports, they required services from a range of providers including physical and mental healthcare, senior centers, legal services, emergency services, and case management. Relatively little is known about service needs among victims of mistreatment. Ayalon (2014) has been the only researcher identifying unmet needs among victims of mistreatment, specifically focusing on older adults who experienced neglect. The study determined that older adults who were neglected often need supervision, medical services, assistance with assistive devices, household tasks, and transportation. However, more is known about unmet needs of older adults in general. In a study of unmet needs for older adults in primary care, Hoogendijk and colleagues (2014) found that unmet needs were more prevalent in the psychological domain (e.g., few social contacts, loneliness, social isolation, difficulty with regular daytime activities) than in physical and environmental domains. Another study among older adults receiving home care also demonstrated unmet needs in social activities, mostly involving interpersonal relationships and community life (Turcotte et al., 2015). Thus, it is likely that elder mistreatment victims also have unmet psychological or social needs before the COVID-19 pandemic, which could be exacerbated by the preventative measures for the COVID-19 pandemic (Luchetti et al., 2020).

## Current Study

Meeting mistreatment victims’ service needs is especially critical during the pandemic, since a previous study demonstrated the association between unmet needs and mortality (He et al., 2015). An early study also found elder mistreatment victims mortality rate was already higher, even after health, cognitive status, and depressive symptoms were accounted for (Lachs, et al., 1998). The pandemic likely further challenged victims’ ability to cover their service needs. Therefore, understanding victims’ experiences during the COVID-19 pandemic would allow social and healthcare service providers to work together to address older adults’ social, physical, and mental health necessities, as higher levels of intervention need additional support and coordination between providers.

This study utilized San Francisco APS’ administrative data to examine APS clients’ COVID-19 awareness and unmet needs, including food, medication, medical appointment, and feelings of loneliness. Based on previous studies (Luchetti et al., 2020; Olomi et al., 2019), it is likely that many APS clients had unmet needs and loneliness, though it is unknown how and the extent to which the COVID-19 pandemic has affected these at-risk adults. In addition, since APS services could vary widely (Aurelien et al., 2019), depending on victim characteristics and mistreatment type, unmet needs may vary. For example, neglect victims may have different needs than financial abuse victims. In addition to the common types of mistreatment mentioned (physical, emotional, financial, sexual abuse, neglect, and self-neglect), California APS also investigates isolation, abandonment, and abduction. Therefore, cases included in this study covered a total of nine types of mistreatment.

## Methods

### Procedures

Authorities in San Francisco Bay Area issued a stay-at-home order on March 17, 2020. As one of the first regions in the country to execute the order, San Francisco’s Department of Disability and Aging Services (DAS), the human service agency that coordinates services for older adults, veterans, people with disabilities, and their families, recognized the urgency of supporting clients who might need services during this time, and requested all programs reach out to at-risk clients to offer assistance. As a DAS program, San Francisco APS decided to contact APS clients who had at least one confirmed/inconclusive substantiation of elder mistreatment type in 2020, were above the age of 80, or lived alone. California APS follows the preponderance of the evidence standards, so a confirmed substantiation indicates a 51% or greater probability of supporting the alleged abuse, while an inconclusive substantiation represents a 50% probability that evidence supports some aspects of alleged abuse but does not remove doubt that the abuse occurred.

**Fig. 1:**
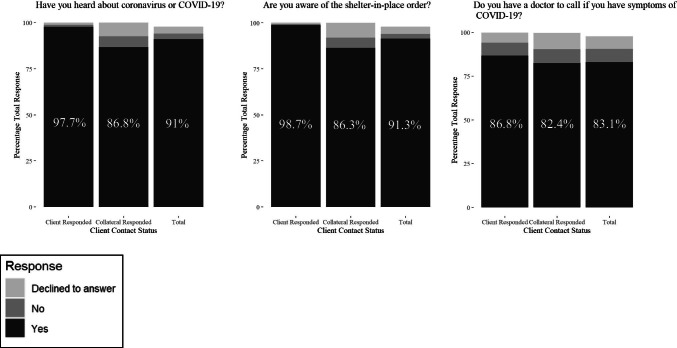
COVID-19 Awareness Percentage by Client Contact Status (Client responded n = 521; Collateral responded n = 393; Total n = 934, including 20 clients who refused to speak with APS before caseworkers asked any questions)

**Fig. 2:**
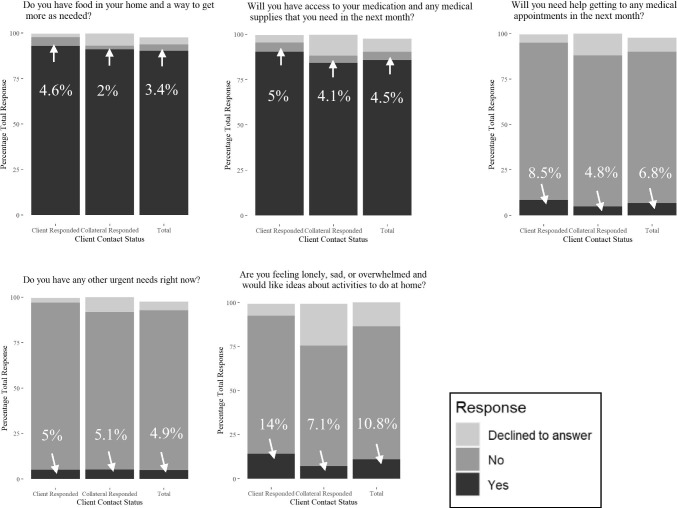
Unmet Needs Percentage by Client Contact Status (Client responded n = 521; Collateral responded n = 393; Total n = 934, including 20 clients who refused to speak with APS before caseworkers asked any questions)

A standardized phone interview protocol was developed by DAS to access clients’ COVID-19 awareness and unmet needs (see Figs. 1 and 2 for questions and response options). APS caseworkers contacted eligible clients and asked questions listed in Figs. 1 and 2, in addition to investigating new mistreatment reports and providing services to ongoing cases. If clients could not be reached via phone after two attempts and leaving a voicemail, APS caseworkers tried contacting a collateral – client’s family, trusted other, or service provider – who was not the alleged abuser. It is not practical to screen for client cognitive impairment during these calls. Since caseworkers have information on client’s ability to consent to services during investigation, they reached out to collaterals if client did not have the ability to consent. When clients/collaterals indicated need and willingness to receive additional support on COVID-19 education or their other unmet needs, even if the unmet needs are not directly asked, APS caseworkers made referrals to a central hub operated by DAS for follow up. Most calls to clients or collaterals lasted between five to 10 min. APS caseworkers documented their phone interviews or attempted calls in a DAS database created for this outreach activity. APS caseworkers could also add optional case notes as part of the phone interview record in the outreach database.

De-identified data shared with Purdue University included APS clients’ demographics and mistreatment substantiation investigated by San Francisco APS between January 1 to March 31, 2020, and the records from phone interviews conducted between March 31 and May 13, 2020. Demographics and mistreatment substantiation were extracted from San Francisco APS’ case management system; phone interviews were extracted from DAS’ outreach database. A unique ID was generated for each case to connect data from the two sources. No consent was obtained since the data were de-identified and obtained directly from San Francisco APS. Institutional Review Board of Purdue University (IRB Protocol # 1812021397) deferred the approval to University of California, San Francisco (IRB # 17-23904) to provide annual oversight.

### Participants

San Francisco APS attempted to contact 1,313 at-risk clients. Among these clients, 1,003 had a confirmed/inconclusive substantiation for at least one type of mistreatment, and 310 were at-risk clients who were over the age of 80 or/and lived alone. One client was excluded from this study due to lack of phone interview records. A total of 934 (71%) clients or their collaterals were reached, while 378 (29%) clients/collaterals could not be reached. Out of the 934 successful contacts, 20 clients declined to speak with APS; 521 clients responded; and 393 collaterals responded. Out of the 378 clients not contacted, 142 received a voicemail; 93 lived in a licensed facility; 71 did not have a voicemail; 38 were deceased; 25 had a wrong phone number; and 9 moved out of county.

### Analysis Plan

Secondary data analysis using mixed methods concurrent design was conducted utilizing San Francisco APS’ mistreatment investigation and phone interview records. For quantitative data analysis, comparison between successful versus unsuccessful contacts were described, as well as successful contacts between clients versus collaterals. In addition to descriptive statistics and Chi-square tests on interview questions and response options between clients versus collaterals and ethnic groups, binary logistic regression with Firth adjusted maximum likelihood estimation method was used to identify predictors of interview questions, including victims’ mistreatment type and age (Firth, 1993). The penalized likelihood estimation was deemed more effective due to quasi-separated data that was present in a large number of regressors. Quantitative data were coded and analyzed using R software version 4.0. Thematic analysis was used on case notes for qualitative data analysis (Braun & Clarke, 2006; Guest et al., 2012), assuming caseworkers’ notes reflected the reality. Qualitative data were independently reviewed by two researchers (P.L. and S.S.) for open coding to generate main and sub-themes. The two researchers met to discuss emergent themes to ensure consistency and resolve discrepancies, then applied the main and sub-themes (see Table 1) in the codebook to the qualitative case notes.

**Table 1:** Themes Presented in the Results

Main themes	Sub-themes	Example quote
1. Client’s preparedness for COVID-19	a. Well-prepared clients	“The client said he has plenty of food and no problem getting more when he needs it. He reports he has all his medications and is able to refill them without any problem. He shared that he is following the social distancing recommendations and the shelter in place order. He knows how to contact his PCP and he has good friends who are supportive and are able to help him if he needs it.”
	b. Clients who were aware of COVID-19 but lack resources	“Case manager stated that despite client knowing about sheltering in place, client is not sheltering in place. Client receives food stamps, and gets food on his own, and also goes to the clinic three times a week to pick up meds. Client doesn’t have a PCP.”
	c. Clients who did not comprehend COVID-19	“She [daughter] did not think client knew about COVID-19 due to her dementia but they have been supervising her and stopping her from going out.”
2. Unmet needs during COVID-19	a. Needs for common supplies, such as food and medications	“Client reported he has food, but very little because he can’t always go out and shop.”
	b. Caregiving needs	“Client is stressed out while taking care of her mother.”
	c. Other needs	“Requested additional financial support.”
3. Loneliness during COVID-19	a. Related to COVID-19 preventative measures	“Client is concerned and overwhelmed about the COVID-19 pandemic.”
	b. Related to mental health history	“Client indicated he feels moody and ‘cursing people out’ because he doesn't have psychiatric meds.”

## Results

Clients/collaterals who were reached were more likely to be females (*χ*^2^ = 3.86, *p* < 0.05) or have a confirmed substantiation for physical abuse (*χ*^2^ = 7.91, *p* < 0.05), compared with those who could not be reached. Compared with clients who had a collateral to respond, clients who responded to the phone interview themselves were more likely to be females (*χ*^2^ = 7.22, *p* < 0.05), White (*χ*^2^ = 23.46, *p* < 0.01) and speaking English (*χ*^2^ = 17.20, *p* < 0.01). Interviews conducted with clients also had a lower proportion of confirmed substantiation for neglect (*χ*^2^ = 15.22, *p* < 0.05) and polyvictimization (*χ*^2^ = 31.45, *p* < 0.01), and a higher proportion of confirmed substantiation for financial abuse (*χ*^2^ = 19.11, *p* < 0.01). Sample sizes were too small to interpret for the other cells with significant statistical results (see Appendix for distribution and statistical results).

### Quantitative Results from Interview Questions

#### Clients’ COVID-19 Awareness and Unmet Needs

Results are demonstrated in Figs. 1 and 2. Among the 934 successful contacts, 850 (91%) indicated clients had heard of COVID-19, 853 (91%) were aware of the stay-at-home order (called shelter-in-place in the Bay Area), and 776 (83%) had a doctor to call in case of COVID-19 concerns. A total of 741 (79%) responded positively to all three questions. Clients were more likely to respond that they were aware of COVID-19 (*χ*^2^ = 41.17, *p* < 0.01) and the stay-at-home order (*χ*^2^ = 55.28, *p* < 0.01), while collaterals (client’s family, trusted others, or service provider) were more likely to state clients were not aware of either, or decline responding to the questions. Different ethnic groups did not differ in COVID-19 awareness.

A total of 697 (75%) clients had no unmet essential needs. A total of 843 (90%) had food and ways to get more food, 802 (86%) had access to medication, 778 (83%) could get to medical appointments, 821 (88%) had no other urgent needs, and 678 (73%) did not feel lonely. Compared with collaterals, a higher proportion of clients indicated they had access to medication (*χ*^2^ = 17.47, *p* < 0.01), could get to medical appointments (*χ*^2^ = 20.63, *p* < 0.01), had no other urgent needs (*χ*^2^ = 16.74, *p* < 0.01), did not feel lonely (*χ*^2^ = 58.61, *p* < 0.01), and a slightly higher proportion needed food (*χ*^2^ = 16.99, *p* < 0.01). Interestingly, a higher proportion of clients also stated that they needed help to get to medical appointments and felt lonely, sad, or overwhelmed. On the other hand, collaterals were more likely to decline responding to questions on client’s unmet needs and feelings of loneliness. Overall, when client responded, they were more likely to indicate at least one unmet need (*χ*^2^ = 13.51, *p* = 0.02). Among ethnic groups, African Americans were more likely to need help getting to medical appointments (*χ*^2^ = 16.80, *p* = 0.03), and Hispanics were also more likely to report feeling lonely (*χ*^2^ = 15.56, *p* = 0.05).

#### Who was not Aware of COVID-19 and had Unmet Needs?

Using victim’s mistreatment type and age as predictors, binary logistic regression models that were firth adjusted were built with each awareness and unmet need question as dependent variable (see Table 2). Substantiation decision for all types of mistreatment were included in each model to identify the mistreatment type associated with COVID-19 unawareness and unmet needs. Only age was included as a predictor among all demographic variables because other variables had too much missing data.

**Table 2:** Binary Logistic Regression Using Mistreatment Type and Age to Predict COVID-19 Awareness and Unmet Needs

	COVID-19 Awareness (n = 822)	Shelter-in-Place Awareness (n = 821)	Having a Doctor for COVID-19 (n = 790)	Food Needs Met (n = 819)	Medical Needs Met (n = 789)	Unmet Needs to Medical Appointment (n = 786)	Other Urgent Needs (n = 811)	Loneliness (n = 726)
Self-Neglect	0.41*	0.25**	0.88	1.17	0.64	1.23	1.83	1.32
Neglect	0.39	0.23*	1.53	0.47	0.77	2.12	0.41	0.51
Financial Abuse	4.42*	3.35	0.95	13.78^a^**	1.44	1.12	0.55	0.63
Emotional Abuse	0.94	1.27	0.74	1.40	0.92	0.60	0.69	3.12***
Physical Abuse	3.62^a^	2.27^a^	0.87	0.85	1.03	1.40	1.03	0.41*
Sexual Abuse	0.24^a^	0.17^a^	0.76	0.33	0.30	3.00	3.21	3.24
Isolation	0.21	0.19	1.58^a^	0.63^a^	0.13*	0.58^a^	8.56	1.32
Abandonment	0.86^a^	0.62^a^	0.19	0.59^a^	0.17	0.55^a^	4.21^a^	1.02^a^
Abduction	0.10^a^	0.11^a^	0.19^a^	0.01	0.10^a^	2.83^a^	37.66^a^	N/A
Age	0.97*	0.96*	1.01	1.01	1.01	1.00	1.00	0.99
Constant	493.31***	1710.74***	5.52**	9.36**	12.94***	0.05***	0.04***	0.24**

Note. * p < .05, ** *p* < .01, *** *p* < .001

^a^Quasi-separation of points. N/A: Complete-separation of points, such that no one responded to have been abused. Exp(B) value in each cell represents odds ratio. Mistreatment substantiation decision was coded as 1 = confirmed or inconclusive, 0 = unfounded, undetermined, no allegation

For COVID-19 awareness, older persons (*χ*^2^ = 5.03, *p* = 0.02) and self-neglectors (*χ*^2^ = 4.68, *p* = 0.03) were less aware of COVID-19, but victims of financial abuse were more aware of COVID-19 (*χ*^2^ = 4.32, *p* = 0.04). Older persons (*χ*^2^ = 5.70, *p* = 0.02), self-neglectors (*χ*^2^ = 9.02, *p* < 0.01), and victims of neglect (*χ*^2^ = 5.57, *p* = 0.02) were also less aware of stay-at-home order. No predictor was associated with having a doctor for COVID-19.

Regarding unmet needs, victims of isolation were more likely to have medication/medical supplies needs (*χ*^2^ = 4.58, *p* = 0.03). While victims of emotional abuse were more likely to report feeling lonely (*χ*^2^ = 14.12, *p* < 0.001), victims of physical abuse were less likely to report feeling lonely (*χ*^2^ = 4.33, *p* = 0.04). Surprisingly, victims of financial abuse were more likely to have their food needs met (*χ*^2^ = 8.40, *p* < 0.01). No predictor was found to be associated with transportation to medical appointments and other urgent needs.

### Qualitative Results from Case Notes (see Table 1 for Example Quotes)

#### Theme 1: Clients’ Preparedness for the COVID-19 Pandemic

Qualitative case notes revealed three distinct groups of clients. The first group, well-prepared clients, were aware of COVID-19 and had no unmet needs. “*Client reports he is sheltering in place. He is in contact with his primary care physician *via* the phone. He is getting food delivered to him. He has caregiver services in place. His medications are being mailed to him from [local drug store], and his physical therapist is coming to his home to give him physical therapy each week.*” Some clients received meals-on-wheels delivery, and a few mentioned they connected with others via internet. Almost all of these clients mentioned having assistance from family, friends, caregivers, or service providers, and they fared well since the support was in place and not interrupted.

In the second group, clients were aware of COVID-19 but had to leave their home at least occasionally. Most of these clients stated that they need to purchase food and get medication, “*I live by myself, I have to eat, I put a mask on and go myself.*” Some clients told APS caseworkers that online grocery delivery’s capacity was limited, while others could only use their electronic benefit transfer in certain stores that did not deliver. A few clients were homeless or lived in a hotel. Additionally, some of clients did not have access to facial masks. “*Client did ask if we were able to provide masks,*” indicating awareness of COVID-19, but lack of resources. Some of these clients might need help if the pandemic does not come to an end soon, “*client has food at home right now, but it’s getting harder for her to order grocery delivery or ask her neighbors/friends to help out on a regular basis.*”

The last group of clients was not able to comprehend COVID-19 or the stay-at-home order. Collaterals told APS caseworkers that clients had dementia, “*Client needs a lot of assistance with personal care and needs to be monitored due to trying to go outside on his on and does not understand shelter-in-place.*” Some clients had severe mental health or substance abuse issues. “*Client has schizophrenia and won’t follow any direction provided to him. Client stays in the streets and hardly in his room except for sleeping. Client also has a payee representative at [name of service provider]. Client is connected to services but lacks awareness of his situation due to his mental health.*”

#### Theme 2: Unmet Needs during the COVID-19 Pandemic

While well-prepared clients fared well, the latter two groups of clients indicated unmet needs for essentials. Food, medication, and supplies (such as toilet paper and facial masks) were common necessities lacking, even if clients were willing to take the risk and step outside of their homes. “*Client [is] in need of one medication but doctor’s office ‘cannot squeeze her in’ to be seen and be prescribed meds, as stated by caretaker. Client has been out [of medication] for past month. Client also received an eviction notice.*” The need for personal care was also prevalent. Some collaterals, especially caregivers, seemed to be under stress and requested In-Home Supportive Services (IHSS). Other clients simply did not have anyone to help with personal care needs. “*Client and spouse will benefit from IADLs assistance, but they don’t qualify for MediCal/IHSS or affordable support at home due to assets exceeding income requirement for these programs. At the same time they can’t afford private pay home care.*” Other unmet needs mentioned included doing laundry, getting transportation to medical appointments, covering pet supplies, and mitigating eviction risk by paying rent, insurance, or other bills.

#### Theme 3: Loneliness during the COVID-19 Pandemic

Lastly, the stay-at-home order led to feelings of loneliness for some clients. “*He is sad that he now has no visitors due to COVID-19.*” Quite a few clients said they could not attend adult day care programs; or they do not use the internet to connect with others. Aside from the digital divide in learning to navigate the internet, the cost of acquiring a computer and internet was not always feasible. Clients with history of depression were especially impacted, and they mentioned seeking help from psychiatrists and medication. “*Client is greatly concerned with COVID-19. Her psychiatrist is helping her deal with it.*” “*Client was upbeat during our call, but said that she has been sad, depressed, stressed at times, but takes medications, listens to music, has supportive friends and those things have helped her to not feel all alone.*”

## Discussion

### Majority of Participants Experience No Unmet Needs during the COVID-19 Pandemic

This study examined elder mistreatment victims’ awareness of COVID-19 and unmet needs at the beginning of the SARS-COV-2 outbreak in San Francisco, since they were at higher risk for both COVID-19 and elder mistreatment. Generally, three quarters of at-risk APS clients and collaterals interviewed affirmed clients’ awareness of COVID-19 and indicated no immediate unmet needs. Considering most APS clients likely required interventions from one or more service providers specialized in addressing their complex physical and mental health concerns (Yunus et al., 2019), San Francisco APS clients adapted fairly well to the pandemic. Since these victims had their cases investigated by APS and were offered services before the pandemic, clients in this study had the benefit of APS involvement. Thus, many clients had already received services and were connected to a service provider (e.g., IHSS, meals-on-wheels) as indicated in case notes, which brought more stability to their lives.

In addition to service providers, family and friends were important resources in the pandemic for clients. Not only did some clients rely on people they trusted to meet essential needs, such as picking up food and medication, their personal care was also fulfilled by these family and friends. Since case notes revealed a few clients could benefit from additional personal care, possible caregiver burnout could be a concern over the length and intensity of pandemic and the associated increased anxieties and financial burden (Beach et al., 2021; Makaroun et al., 2020). This is especially true with family caregiver collaterals, who often told APS caseworkers that clients had cognitive impairment and were unaware of COVID-19 and the stay-at-home order, because theses clients’ levels of care were likely higher.

### Unmet Needs and Loneliness during the Pandemic

Nonetheless, a quarter of APS clients and collaterals reported at least one unmet needs. Some of their unmet needs were a result of the onset of the pandemic. Older persons, self-neglectors, and victims of neglect were less likely to know about the COVID-19 pandemic or the stay-at home order. Even though most clients indicated awareness of COVID-19 and the stay-at-home order, a lot of them could not stay at home. Some clients did not have help to purchase food or get medication, while some needed to get to medical appointments. For example, victims of isolation had trouble getting access to medication and medical supplies. These activities, though considered essential, increased their risk of contracting COVID-19. Although ethnic differences were not able to get included in statistical modeling due to missing data, descriptive statistics informed us African Americans reported needing help to get to medical appointments, demonstrating higher level of unmet needs among some minorities. Interestingly, victims of financial abuse were more aware of the pandemic and had their food needs met. We suspected that the victimized experience might make them more inclined to ensure necessities are covered.

Feelings of loneliness, as experienced by one in four APS clients, resulted from the measures (i.e., the stay-at-home order) that were intended to slow the spread of COVID-19. Clients were isolated from mandated reporters who were also physically distancing, or resources, such as adult day care programs, that were temporarily closed. Since some of these disadvantaged older adults did not have access to technology to connect to family/friends or service providers to obtain social support and services (Seifert et al., 2020), loneliness became a major negative consequence, especially among those with depression (Ayalon et al., 2020). Additionally, victims of emotional abuse were more likely to report feelings of loneliness. Given that social embeddedness and support has been a consistent protective factor (Pillemer et al., 2016; Roberto, 2016), loneliness and isolation that came with the lockdown increased risk of elder mistreatment among this already victimized population. Since Hispanics were more likely to report feeling lonely, service providers might want to reach out to this historically underserved minority group. Surprisingly, victims of physical abuse reported less loneliness, which could be a sense of relief not having to interact with people who could hurt them.

Compared with unmet services needs reported in Olomi and colleagues' 2019 study, APS clients in our study reported fewer unmet needs. For example, 45% of individuals reported medical needs and 28% reported food needs in their study, but only 5% and 3% in this study. These differences may be the result of differences between the samples. In Olomi et al.’s study, their sample size was 40 long-term elder mistreatment victims who were reported to the police, which were likely to be more severe cases. Our sample size of 934 was much bigger and included 310 at-risk APS clients who had no confirmed/inconclusive findings in elder mistreatment. In addition, unmet needs questions were framed differently. In Olomi et al.’s study, older adults read a list of services, including medical health services, then they were asked “What other services or responses would you like to be available for older adults at risk for maltreatment, neglect, and financial exploitation?” In our study, the medical health question was asked directly.

This is not to say our study, which relied on self-report of clients and collaterals, must have accurately reflected at-risk APS clients’ unmet needs. Population-based studies found victims of elder mistreatment had lower levels of global cognitive function, episodic memory, calculation, perceptual speeds, and executive functioning, compared with other community-dwelling older adults (Wood et al., 2014). Therefore, it is possible that the actual statistics of unmet needs was much higher, or clients could have underestimated when and how much help they might need due to the lack of planning abilities in executive functioning. It is also possible that some clients might be in need of help, but were reluctant to say so as they did not want (or fear) APS involvement. Moreover, collaterals were more likely to decline responding, probably partly because they would like to protect client’s privacy (just like the clients who declined to speak with APS caseworkers), or they could be unclear on clients’ COVID-19 awareness and unmet needs.

Additionally, 29% of total clients were not reached. Deducting the clients who moved to receive higher level of services in licensed facilities and a small number who passed away, close to 20% of total clients could not be contacted. This demonstrates the challenges in serving this at-risk population – they might not have a phone, become homeless without support from collaterals, or have their network cut off by abusers (Olomi et al., 2019). Unmet needs were likely higher among this group. Similarly, if elder mistreatment victims served by APS needed assistance, victims not reported to APS – who have been facing elder mistreatment and COVID-19 during this time – were even more likely to need services, maybe at much higher levels.

### Service Providers’ Role during the Pandemic

Case notes informed us some clients with unmet needs required immediate help to acquire food, medication, or supplies. These complex or severe cases call for increased involvement of multiple service providers. One previous study found that integrated-service-delivery networks led to fewer unmet needs among disabled older persons with home-care needs in the community (Dubuc et al., 2011), and an integrated social and health services system could be especially important to cover needs of elder mistreatment victims during crises. Since this population is at-risk of COVID-19, service providers need to focus on assisting with essential activities during the pandemic, such as getting food and medication, or arranging for medical transportation to reduce COVID-19 risk. In addition, since this population is also at-risk of elder mistreatment, healthcare providers who are able to provide routine care or telehealth have a unique opportunity to decrease elder mistreatment risk for this population. (Makaroun et al., 2020).

Since the AIM called on APS to serve this at-risk population (Han & Mosqueda, 2020), San Francisco APS demonstrated one of the ways APS programs could serve the most at-risk clients. Calling clients was just the first step to detect client’s unmet needs. APS caseworkers provided resources needed by clients. For example, one of the San Francisco APS supervisors located a person who was making cloth masks to distribute to those who requested them. San Francisco APS has been able to offer surgical masks when they visit a client if the client is not wearing a mask and agrees to take a mask. Some services could not be fulfilled by APS caseworkers. In such cases, the DAS followed up with clients in need of services to connect them with service providers. Obviously, teamwork and collaboration between APS and service providers is the key to serve these clients. In San Francisco, collaboration between APS, IHSS, and two community agencies responsible for caregiving and care coordination, has been taking place in a special program in response to the COVID-19 pandemic. This team serves at-risk adults who were moved from San Francisco’s shelters, streets, hospitals, and nursing homes to shelter-in-place (SIP) sites as the result of the COVID-19 pandemic. Many of these adults require an array of coordinated supportive services, and the team assists them to obtain services needed to live safely at the SIP sites. In the spirit of self-determination in adult safeguarding, services offered follow clients’ will and respect their autonomy.

## Limitations and Future Research

The phone interview protocol was not designed for research. Therefore, not all questions were ideal for research. For example, the unmet need questions on food asked about having enough food and having ways to get more food, which was double-barreled. Also, the loneliness question asked about having any of the three negative feelings (lonely, sad, or overwhelmed) and willingness to receive activity ideas. Since clients likely would indicate unmet needs only if they agreed with both statements, the actual level of unmet needs might be higher. In addition, since the study is cross-sectional and captured the early views of APS clients, the ongoing nature of this pandemic would likely produce more significant unmet needs. Future studies should focus on at-risk clients’ unmet need across time to investigate the resources clients might have drawn or lack during crisis. Nonetheless, San Francisco APS concluded the wellness check in December 2020, a few weeks after the second stay-at-home order was issued in the end of November 2020, because clients’ unmet needs seemed to dissipate.

Moreover, San Francisco APS is relatively well-funded compared with other APS programs. APS does not have dedicated federal funding, and programs operate at county or state level (Liu & Ross, 2021). A recent publication further described the challenges APS faced that are brought forth by the pandemic (Liu & Delagrammatikas, 2021). It is unclear if other APS programs in the country have the resources to check on past clients and provide services, or if local service providers are able to continue supporting victims during the pandemic. This urban community has more resources to offer when clients are willing to accept assistance, but rural communities in the country might not have the same level of resources. Using findings from this study to advocate for funding, APS programs could explore opportunities to partner with other agencies, such as healthcare services, public health department, Area Agency on Aging, law enforcement, or non-profit community agencies, who are also concerned with at-risk adults to pool resources together to serve this vulnerable population more effectively. Forming a taskforce early with potential partners not only helps respond to the COVID-19 pandemic, but also with other crises such as wildfires, excessive smoke, heat, cold, and power outages. If possible, a statewide initiative could assist smaller counties that have less resources to establish a local coalition, or facilitate a regional approach for smaller counties to support each other, or to join a larger county for combined outreach effort.

## Conclusion

During this time of uncertainty, research on elder mistreatment victims helps quantify the potential impact of the COVID-19 pandemic on one of the most vulnerable populations at risk during the pandemic. In many instances, these at-risk adults’ needs were not fully met by social and healthcare services, or they could have refused to accept assistance when APS was first involved. Wellness checks could lead to the discovery of systematic gaps for obtaining necessities or client’s change of heart to receive help, especially during crises like the pandemic. If this population’s unmet needs could be detected early, negative consequences often faced by this population could be prevented, including homelessness, exacerbation of mental health issues, emergency department usage. These APS clients will require on-going wellness checks because many may run out of supplies or care resources as the pandemic continues. Collecting data about the impact of the COVID-19 pandemic on this population may provide insights about the condition of those who have contracted or recovered from the disease, the impact of mitigation strategies on the well-being of this population, and informed responses to future public health crises.

## Appendix

See Table 3.

**Table 3:** Demographics and Mistreatment Type by Client Contact Status

		Not Contacted (n = 378)		Contacted (n = 934)		Statistics	*p*-value	Client Declined (n = 20)		Client Responded (n = 521)		Collateral Responded (n = 393)		Statistics	*p*-value
Demographics															
Age			71.46		70.30	1.23^a^	0.22		73.16		72.01		70.67	0.95^c^	0.39
Gender	Female	170	47.22%	465	53.57%	3.86^b^	0.05	11	61.11%	272	57.38%	182	48.40%	7.22^b^	0.03
	Male	190	52.78%	403	46.43%			7	38.89%	202	42.61%	194	51.60%		
Education Level	More than High School	8	21.05%	29	30.21%	1.16^b^	0.56	0	0.00%	19	35.19%	10	24.39%	4.17^b^	0.38
	High School	17	44.74%	39	40.63%			0	0.00%	22	40.74%	17	41.46%		
	Less than High School	13	34.21%	28	29.17%			1	100.00%	13	24.07%	14	34.15%		
Race and Ethnicity	White	150	52.26%	343	49.78%	2.79^b^	0.59	2	18.18%	210	56.15%	131	43.09%	23.46^b^	< 0.01
	African American	40	13.94%	110	15.97%			1	9.09%	53	14.17%	56	18.43%		
	Hispanic	43	14.98%	88	12.77%			4	36.36%	44	11.76%	40	13.16%		
	Asian	46	16.03%	132	19.16%			3	27.27%	58	15.51%	71	23.36%		
	Other	8	2.79%	16	23.22%			1	9.09%	7	2.41%	4	1.97%		
Language Type	English	289	79.61%	653	74.20%	5.42^b^	0.14	8	71.65%	372	77.43%	273	71.65%	17.20^b^	0.01
	Spanish	16	4.41%	55	6.25%			3	6.56%	27	5.61%	25	6.56%		
	Asian Languages	41	11.29%	134	15.23%			3	17.65%	63	13.10%	68	17.85%		
	Other Languages	17	4.68%	38	4.31%			3	17.65%	19	3.95%	15	3.94%		
Speaks English	Yes	325	86.67%	745	82.59%	2.94^b^	0.09	18	90.00%	414	83.30%	313	81.30%	1.38^b^	0.50
	No	50	13.33%	157	17.41%			2	10.00%	83	16.70%	72	18.70%		
Marital Status	Never Married / Single	133	59.91%	305	52.31%	4.66^b^	0.10	4	40.00%	162	52.09%	139	53.05%	2.71^b^	0.61
	Divorced / Separated / Widowed	55	24.77%	155	26.59%			4	40.00%	88	28.30%	63	24.05%		
	Domestic Partnership / Married	34	15.32%	123	21.10%			2	20.00%	61	19.61%	60	22.90%		
Mistreatment Type															
Self-Neglect	Confirmed	168	99.40%	337	99.12%	0.50^b^	0.78	2	100.00%	154	98.09%	181	100.00%	3.53^b^	0.47
	Inconclusive	0	0.00%	1	0.29%			0	0.00%	1	0.63%	0	0.00%		
	Unfounded	1	0.59%	2	0.59%			0	0.00%	2	1.27%	0	0.00%		
	Undetermined	0	0.00%	0	0.00%			0	0.00%	0	0.00%	0	0.00%		
Neglect	Confirmed	11	26.19%	27	25.47%	2.88^b^	0.41	0	0.00%	7	16.28%	20	32.79%	15.22^b^	0.02
	Inconclusive	11	26.19%	36	33.96%			0	0.00%	15	34.88%	21	34.43%		
	Unfounded	15	35.71%	38	35.85%			1	50.00%	18	41.86%	19	31.11%		
	Undetermined	5	11.90%	5	4.72%			1	50.00%	3	6.98%	1	1.64%		
Financial Abuse	Confirmed	36	39.56%	115	47.52%	4.83^b^	0.18	6	54.55%	81	55.86%	28	32.56%	19.11^b^	< 0.01
	Inconclusive	28	30.77%	67	27.69%			3	27.27%	34	23.45%	30	34.88%		
	Unfounded	17	18.68%	48	19.83%			1	9.09%	28	19.31%	19	22.09%		
	Undetermined	10	10.99%	12	4.96%			1	9.09%	2	1.38%	9	10.47%		
Emotional Abuse	Confirmed	21	33.87%	90	46.63%	4.25^b^	0.24	2	40.00%	62	49.60%	26	41.27%	5.82^b^	0.44
	Inconclusive	21	33.87%	50	25.91%			1	20.00%	33	26.40%	16	25.40%		
	Unfounded	15	24.19%	45	23.32%			1	20.00%	25	20.00%	19	30.16%		
	Undetermined	5	8.06%	8	4.15%			1	20.00%	5	4.00%	2	3.17%		
Physical Abuse	Confirmed	17	39.53%	57	55.34%	7.91^b^	0.05	1	50.00%	36	59.02%	20	50.00%	2.08^b^	0.91
	Inconclusive	14	32.56%	17	16.50%			0	0.00%	10	16.39%	7	17.50%		
	Unfounded	7	16.28%	24	23.30%			1	50.00%	12	19.67%	11	27.50%		
	Undetermined	5	11.63%	5	4.85%			0	0.00%	3	4.92%	2	5.00%		
Sexual Abuse	Confirmed	1	20.00%	6	46.15%	5.18^b^	0.16	0	0.00%	3	42.86%	3	50.00%	NA	NA
	Inconclusive	3	60.00%	4	30.77%			0	0.00%	1	14.29%	3	50.00%		
	Unfounded	0	0.00%	3	23.08%			0	0.00%	3	42.86%	0	0.00%		
	Undetermined	1	20.00%	0	0.00%			0	0.00%	0	0.00%	0	0.00%		
Isolation	Confirmed	1	16.67%	3	15.00%	8.78^b^	0.03	0	0.00%	1	7.69%	2	28.57%	NA	NA
	Inconclusive	0	0.00%	7	35.00%			0	0.00%	6	46.15%	1	14.29%		
	Unfounded	3	50.00%	10	50.00%			0	0.00%	6	46.15%	4	57.14%		
	Undetermined	2	33.33%	0	0.00%			0	0.00%	0	0.00%	0	0.00%		
Abandonment	Confirmed	0	0.00%	3	37.50%	1.41^b^	0.50	0	0.00%	0	0.00%	3	37.5%	NA	NA
	Inconclusive	0	0.00%	2	25.00%			0	0.00%	0	0.00%	2	25.00%		
	Unfounded	1	100%	3	37.50%			0	0.00%	0	0.00%	3	37.50%		
	Undetermined	0	0.00%	0	0.00%			0	0.00%	0	0.00%	0	0.00%		
Abduction	Confirmed	0	0.00%	1	33.33%	2.22^b^	0.33	0	0.00%	0	0.00%	1	33.33%	NA	NA
	Inconclusive	1	50.00%	0	0.00%			0	0.00%	0	0.00%	0	0.00%		
	Unfounded	1	50.00%	2	66.67%			0	0.00%	0	0.00%	2	66.67%		
	Undetermined	0	0.00%	0	0.00%			0	0.00%	0	0.00%	0	0.00%		
Polyvictimization	0 Type	87	23.01%	222	23.77%	5.76^b^	0.45	6	30.00%	129	24.76%	87	22.14%	31.45^b^	< 0.01
	1 Type	139	36.77%	388	41.54%			10	50.00%	245	47.02%	133	33.84%		
	2 Types	82	21.69%	192	20.56%			4	20.00%	89	17.08%	99	25.19%		
	3 Types	44	11.64%	88	9.24%			0	0.00%	40	7.67%	48	12.21%		
	4 Types	20	5.29%	32	3.43%			0	0.00%	13	2.49%	19	4.83%		
	5 Types	4	1.06%	9	0.96%			0	0.00%	3	0.58%	6	1.52%		
	6 Types	2	0.53%	3	0.32%			0	0.00%	2	0.38%	1	0.25%		

^a^t-test.

^b^Chi-square.

^c^ANOVA
